# Effect of Porosity/Binder Index on Strength, Stiffness and Microstructure of Cemented Clay: The Impact of Sustainable Development Geomaterials

**DOI:** 10.3390/ma17040921

**Published:** 2024-02-17

**Authors:** Jair de Jesús Arrieta Baldovino, Yamid E. Nuñez de la Rosa, Oriana Palma Calabokis

**Affiliations:** 1 Civil Engineering Program, Universidad de Cartagena, Cartagena de Indias 130015, Colombia; 2 Faculty of Engineering and Basic Sciences, Fundación Universitaria Los Libertadores, Bogota 110231, Colombia; opalmac@libertadores.edu.co

**Keywords:** soil cementation, tropical geomaterials, sustainable construction, waste materials, geotechnical risk mitigation

## Abstract

Searching for alternative material options to reduce the extraction of natural resources is essential for promoting a more sustainable world. This is especially relevant in construction and infrastructure projects, where significant volumes of materials are used. This paper aims to introduce three alternative materials, crushed ground glass (GG), recycled gypsum (GY) and crushed lime waste (CLW), byproducts of construction industry geomaterials, to enhance the mechanical properties of clay soil in Cartagena de Indias, Colombia. These materials show promise as cementitious and frictional agents, combined with soil and cement. Rigorous testing, including tests on unconfined compressive strength (*q*_u_) and initial stiffness (*G_o_*) and with a scanning electron microscope (SEM), reveals a correlation between strength, stiffness and the novel porosity/binder index (*η*/C*_iv_*) and provides mixed design equations for the novel geomaterials. Micro-level analyses show the formation of hydrated calcium silicates and complex interactions among the waste materials, cement and clay. These new geomaterials offer an eco-friendly alternative to traditional cementation, contributing to geotechnical solutions in vulnerable tropical regions.

## 1. Introduction

When the implementation of engineering work is necessary, one of the main aspects to be analyzed is the load capacity of the soil mass that supports that structure. Sometimes, the geotechnical engineer faces soils with low bearing capacity, identified through geotechnical investigations, which allow analyzing regions, layers or soil masses with resistance and deformability characteristics unfavorable to the intervention intended to be carried out on the terrain [1]. In these situations, three distinct alternatives can be adopted to implement the project: replacing the inadequate soil layer with a material with better properties, adapting the project to the properties of the local soil and applying improvement techniques that aim to change the soil properties [2]. Generally, the first two options are not economically viable solutions to implement, considering that the replacement of the inadequate soil layer is easily carried out up to depths of three to four meters, and beyond that, additional costs may be incurred with borrowing operations. Adapting the project to the characteristics of the material can lead to expenses incompatible with the budget available for the project’s construction, as the solution usually involves using deep foundations [3]. Considering this, improving the local soil can be an excellent cost–benefit alternative. There are various technologies applicable to soil improvement, which are grouped into two main types of interventions: terrain reinforcement and soil treatment [4].

The chemical treatment technique consists of adding a specific chemical substance to the soil to induce changes that affect its mechanical strength, permeability and deformability properties, thereby achieving the goal of improvement. Chemical treatment can be applied to various types of soils, from soft clays to weak rocks. The most used binders are Portland cement, lime, blast furnace slag, fly ash and silica fume. Binders derived from industrial byproducts can act in conjunction with Portland cement or lime, independently through pozzolanic reactions or alkali-activated with alkaline solutions [5].

The material resulting from the soil–cement technique is different from concrete in various aspects. One of the main differences is that, in concrete, there is a quantity of paste (cement + water) sufficient to coat the surface of the aggregates present in the mix and fill the existing voids. In soil–cement mixtures, however, the amount of paste is insufficient to cover the surface of all soil particles and fill the existing voids, resulting in a cementitious matrix that binds together nodules of uncemented aggregates [6].

In this context, alternative materials such as glass, gypsum and limestone often emerge as byproducts of industry, construction and demolition. Various studies have been conducted using various types of cement in geotechnical problems. When well proportioned, these types of cements can exhibit properties such as a cementitious matrix with good strength and durability and reduced porosity. Beyond the physical performance of the material, another issue raised is the destination of industrial byproducts, which often generate various environmental problems. These problems can be avoided by allocating these materials for appropriate purposes [7,8].

Generally, it is sought to compact improved soils with a pre-determined moisture content, as the amount of water influences the achievement of a certain maximum dry specific gravity, and this value corresponds to a single moisture content (optimum moisture content). In soil–cement mixtures, a minimum water content must be ensured for the total hydration of the cement. Thus, more significant quantities require a higher moisture content [9]. If the moisture during the compaction of soil cement is higher than the minimum amount, the excess water exhibits a deleterious behavior for developing strength. Soil stabilization techniques have been historically significant for the construction industry, for improving soil strength and for managing its properties. One innovative method explored is using glass powder in soil stabilization. Using glass powder, gypsum residues and limestone waste, often derived from recycled materials, presents both environmental and structural advantages [10].

For GY uses in soil stabilization, Edora [11] investigated the effects of adding gypsum and rice husk ash on expansive soil’s resistance and permeability characteristics. The results indicated that the incorporation of gypsum and rice husk ash into the expansive soil generated notable improvements in its resistance and permeability. Likewise, an increase in the compressive and shear resistance of the stabilized soil was observed. Degirmenci et al. [12] verified that adding gypsum, cement and fly ash can reduce the plasticity of soils and improve their resistance and compaction characteristics. Yilmaz and Civelekoglu [13] also investigated the effects of gypsum on stabilizing clay soils and analyzed its impact on reducing soil expansion and contraction. Ahmed and Ugai [14] investigated the environmental durability of soils stabilized with recycled gypsum. Although environmental factors such as temperature and humidity affect the durability of soils stabilized with recycled gypsum, this can be solved by adding additives and modifiers, such as cement and fly ash. The authors concluded that these additives help increase the strength and stability of soils against adverse environmental effects.

Integrating glass powder in soil stabilization practices is a multifaceted exploration aimed at sustainable construction methodologies. In the quest for environmentally friendly alternatives, researchers have investigated the application of soda lime glass powder (SLGP) for expansive soil stabilization, highlighting its potential to mitigate the challenges posed by soil expansiveness [15]. Simultaneously, studies have examined the use of waste glass powder to stabilize high-plasticity clay in Erbil City, Iraq, addressing the specific engineering challenges associated with local soil properties [14]. Expanding the horizon of innovation, using glass powder and silica fume in sugarcane bagasse ash-based geopolymers was explored for soil stabilization, offering a holistic approach combining various materials for enhanced effectiveness [16]. Furthermore, the exploration extends to developing eco-friendly pavements manufactured with glass waste, focusing on the physical and mechanical characterization of these pavements and their applicability in soil stabilization [17]. Collectively, these investigations contribute valuable insights into sustainable practices that amalgamate glass-based materials with traditional soil stabilization techniques, paving the way for eco-conscious and practical solutions in construction.

When ground into a fine powder, recycled glass or ground glass (GG) has pozzolanic properties. This means it can react with calcium hydroxide in the presence of moisture to form a cementitious compound [18]. The glass powder can enhance the soil’s binding properties when mixed with soils, especially clayey soils. This improvement can lead to increased shear strength, reduced shrink–swell potential and more excellent resistance to erosion. From an environmental perspective, using glass powder in soil stabilization promotes recycling and waste reduction. Every ton of recycled glass reduces the need to extract and process raw materials, leading to energy savings and reduced carbon emissions. Moreover, repurposing waste glass can mitigate landfill challenges [19]. However, there are challenges and considerations to keep in mind. The particle size of the glass powder, its specific surface area and the percentage of its incorporation into the soil can significantly influence the final properties of the stabilized soil. Ensuring an optimal mixture is crucial for achieving the desired results [20].

Crushed limestone waste (CLW) and recycled gypsum (GY) are two alternative residues for soil stabilization that have been used in recent studies [13,21]. Gypsum in fine particles can help increase the resistance of the soil in a short time, acting as a catalyst. The gypsum in cement and lime can accelerate the hydration reaction and produce more ettringite, further enhancing the stabilized soil strength. On the other hand, stone dust acts as artificial sand, giving more friction to the mixture and increasing the soil’s mechanical resistance.

When using the soil–cement technique, the aim is to determine a mixture that, when implemented, meets the minimum requirements of mechanical characteristics such as strength, stiffness and durability. The resistance of soils improved with Portland cement is directly influenced by porosity and the volume of the cement. Porosity, referring to empty spaces in the soil, decreases as cement fills these spaces, creating a more compact and resistant matrix. Lower porosity contributes to higher resistance, reducing susceptibility to compression and deformation. Additionally, the amount of cement plays a crucial role; a higher content is generally associated with greater strength as long as a proper balance is maintained to avoid excessive shrinkage. The water/cement ratio is also essential to achieving a workable mix and facilitating a chemical reaction that strengthens the soil. Optimizing porosity and the amount of cement in the mix is fundamental to achieving high-strength and durable soils improved with Portland cement [22,23].

The dosing of soil–cement cannot be based solely on the water/cement ratio, developed for concrete, because the compaction of soil–cement does not promote the complete expulsion of air from the mix, making the filling of voids with water inefficient, unlike what occurs in mortars and traditional concretes. Therefore, the strength of the improved soils cannot be correlated to the water/cement factor [24]. In this way, various studies have been developed to establish a relationship for soil–cement that sets the minimum amount to meet the desired properties, mainly strength and durability. Consoli et al. [25] proposed a rational dosing method for soil–cement mixtures. The authors demonstrated that the unconfined compressive strength (*q*_u_) of cemented soil can be determined by the quotient *η*/$C_{\mathrm{iv}}^{x}$. This parameter is called the voids/cement factor and relates the porosity of the compacted mixture (*η*) to the volumetric content of cement $C_{\mathrm{iv}}^{x}$ adjusted by the internal exponent *x*. Thus, this relationship makes it possible to determine the power–type relation indicated by Equation (1).

(1) $$q_{u}=A_{i}{\left[\frac{\eta }{{\left(C_{\mathrm{iv}}\right)}^{x}}\right]}^{-B}$$

The constant $A_{i}$ in Equation (1) is of significant theoretical importance, as it depends on various factors that influence the strength behavior of the soil–cement mixture. These factors include the soil’s critical state strength ratio, the cement’s uniaxial compressive strength, the cement stress ratio, the porosity at a critical state and the ratio between the unconfined compression and extension strengths [26]. The exponents −*B* and *x* play a vital role in characterizing the relationship between the soil and cement properties and the strength behavior of the mixture. Diambra et al. [27] studied the above-described relationship and observed that the values of *x* and B predominantly depend on the soil characteristics. *x* is approximately the inverse of B (*x* ≈ 1/B). Moreover, the scalar $A_{i}$ is governed by the properties of both the soil and the cementitious matrix. Scheuermann Filho [28] described that the exponent *x* aligns the relationship between porosity and the volumetric content of cement in each soil. If *x* is equal to one (*x* = 1), it indicates that both the porosity and the amount of cement have an equivalent influence concerning the strength of a particular soil mixed with cement. A positive value of *x* less than one (*x* < 1) indicates that the porosity has a greater relevance in the compacted soil–cement mixture. However, if *x* is more significant than one (*x* > 1), the cementitious bonds significantly influence the strength. The method involves developing the voids/cement factor curve versus simple compressive strength, called the ‘dosage curve’. Once the curve is established, the graph determines the voids/cement factor corresponding to the desired simple compressive strength. From this, it is possible to alter the specific weight at which the mixture should be compacted and the amount of cement added, allowing for a balance that minimizes costs.

Furthermore, various studies have explored the possibility of correlating the quotient *η*/$C_{\mathrm{iv}}^{x}$ with other geotechnical parameters of artificially cemented soils, such as the durability, which can be measured and related to the cumulative mass loss. However, few studies have related the porosity/cement factor to adding limestone, gypsum and ground glass waste in the same soil. Therefore, this study aimed to study the relationship between the porosity/binder and clay’s compressive strength, stiffness and microstructure stabilized with more sustainable recycled materials: GG, GY and CLW. For this, waste percentages from 10% to 30%, curing times of 7 and 28 days and Portland cement of up to 6% by weight were used. To verify the porosity/binder’s effectiveness, the compacted mixtures’ dry density was varied according to the results of the Proctor test.

## 2. Experimental Program

**Figure 1:**
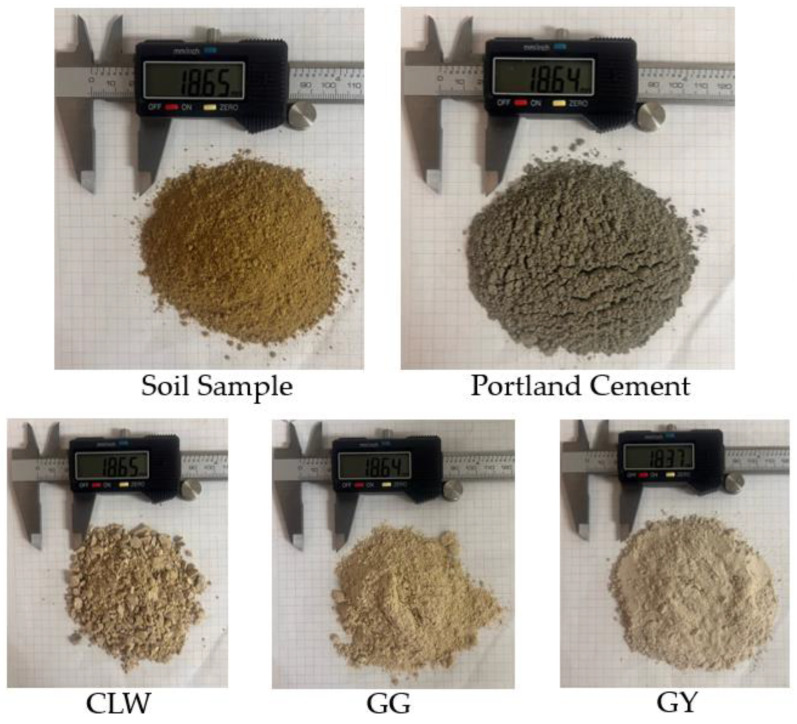
Photo of the raw materials.

The experimental program consisted of 3 stages. The first stage involved collecting, preparing and characterizing all the materials used, i.e., clay, glass waste, gypsum and limestone (Figure 1). The characterization tests included the particle size distribution of the material, its chemical composition, microstructure and plastic properties (for the clay) and the specific gravity of each raw material. The second stage involved preparing compacted clay specimens with each added material combination (GG, GY or CLW) and curing them in a moist chamber. The final stage consisted of conducting non-destructive ultrasound tests to determine the unconfined compressive strength and microstructural properties of the new geomaterials.

The definition and characterization of the materials, as well as the methods, are described below.

### 2.1. Materials

**Figure 2:**
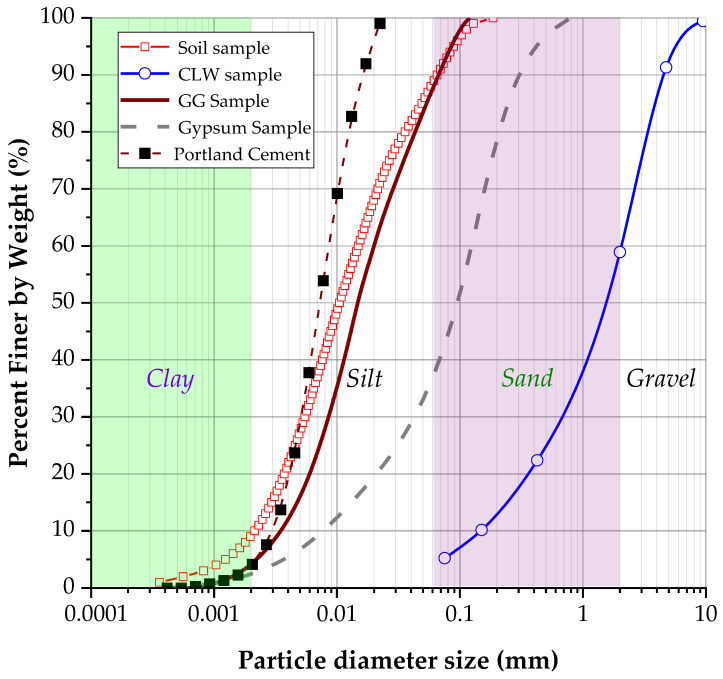
Granulometric curve of the soil sample, CLW, GG, GY and Portland cement.

In this research, the following materials were employed: low-plasticity clay, crushed limestone waste, gypsum residue from plasterboard, ground glass residue, Portland cement and water. The soil was extracted from the northern area of Cartagena de Indias (Colombia; coordinates: 10.537000, −75.462016). The clay belonged to coastal soil deposits, with a moderate potential threat of erosion, mass movement, landslides and moderate dispersal behavior [29]. After field collection, the soil was characterized through laser granulometry tests, specific gravity measurement, Atterberg limits determination, chemical composition analysis and microstructural properties assessment. The results of the soil characterization tests are presented in Table 1. Additionally, Figure 1 presents a photo of the soil, and Figure 2 displays the soil granulometry curve. According to the Unified Soil Classification System (USCS), the clay was classified as low-compressibility clay (named CL). Skempton’s [30] colloidal activity of clay was calculated as 1.60.

**Figure 3:**
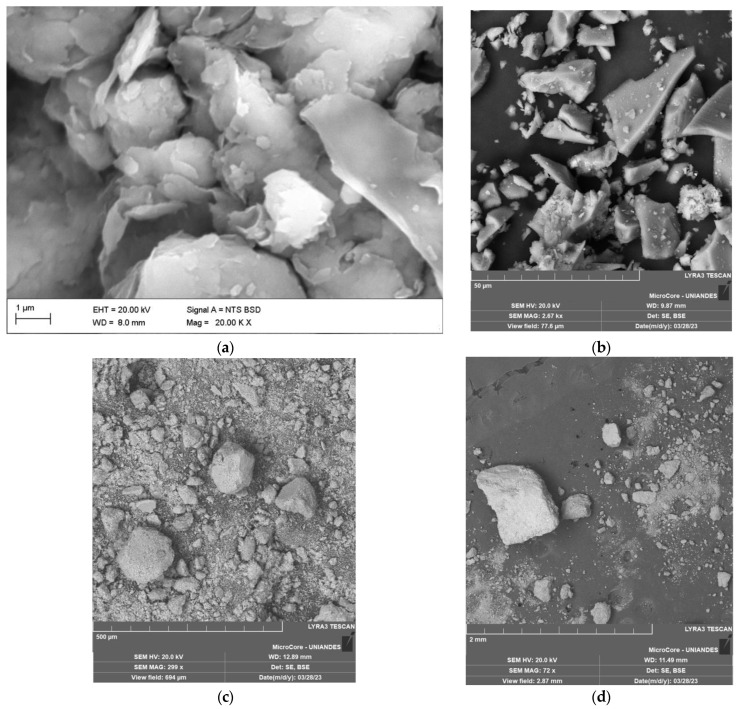
Microstructure of raw materials: (**a**) soil sample; (**b**) GG; (**c**) GY; (**d**) CLW.

The chemical compositions of the soil and raw materials (Figure 1) were determined using X-ray fluorescence (XRF) spectrometry with an EDX-720/800HS spectrometer (Shimadzu company, Tokyo, Japan). XRF is a technique that combines qualitative and quantitative analysis, utilizing the fundamental parameters method based on dispersive energy. XRF analysis was performed under a vacuum, and samples were excited with a rhodium tube and an electrical voltage of 50 kV and 100 Μa. Table 2 provides the soil’s chemical composition obtained through X-ray fluorescence. The soil was composed mainly of kaolinite clay minerals, as presented in the microstructure photo in Figure 3a and studied previously by Acuña et al. [29]. The extracted soil sample sodium levels ranged from 0.72% to 1.94%, and the soil had an optimal moisture content of 26% and a maximum apparent dry unit weight of 13.87 kN/m^3^. Like the clay studied herein, the soils of the northern area of Cartagena de Indias present a slight to moderate dispersivity level.

The CLW was collected from a rock quarry near Cartagena. The residue was characterized through sieving granulometry, specific gravity, chemical composition and microstructural analysis. Figure 2 shows the granulometric curve of the residue, and the physical properties are presented in Table 1. Table 2 displays the chemical composition of the limestone. According to the USCS (Unified Soil Classification System), the limestone is classified as SW. The gypsum powder residue (GY) was obtained from grinding gypsum sheets using a planetary mill. An amount of 40 kg of GY material was collected from demolition waste. An amount of 4 kg of material was placed in the ball mill for approximately 40 min with 1 Hz. After grinding, the residue was sieved through a 1 mm sieve. The chemical composition of the gypsum is presented in Table 2, and its granulometric curve is displayed in Figure 2. According to the USCS, the GY is classified as SW (well-graded fine sand).

The glass powder (mainly composed of silicon) was obtained from grinding glass bottles collected at recycling points in Cartagena. Approximately 40 kg of GY material was collected from demolition waste. The material was ground for 40 min in a ball mill with 1 Hz and was sieved through a 0.15 mm sieve. The chemical composition of the glass is shown in Table 2, and the granulometric curve is shown in Figure 2. Microscopy tests are displayed in Figure 3. According to the USCS, the GY is classified as ML (silt). Figure 3b shows the microstructure of the GG particles. It is noted that the morphology of the particles is irregular and more diminutive than 0.10 mm in diameter. A distinctive structure was revealed when the GG, GY and CLW samples were examined under a microscope with different magnifications. These samples exhibited a disaggregated arrangement, characterized by a formation primarily consisting of grains. Most notably, at this level of magnification, it became apparent that there was no significant chemical interaction between the grains.

Last, the water used in this study came from the local aqueduct source.

### 2.2. Specimen Molding and Preparation

**Figure 4:**
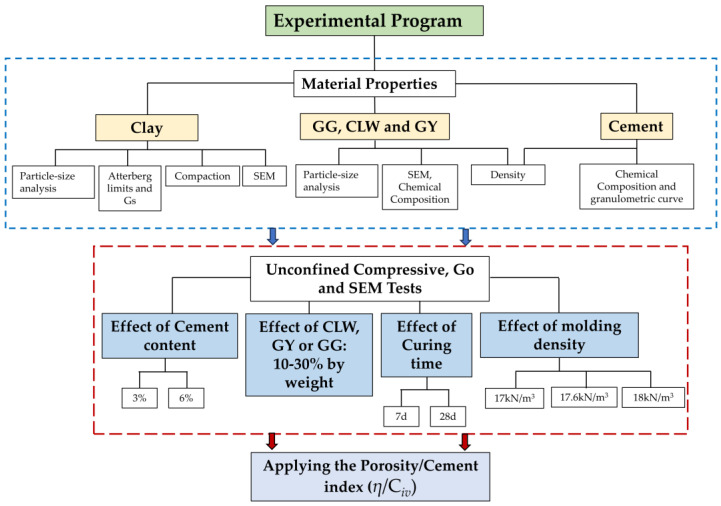
Flowchart of the experimental program.

Figure 4 offers a comprehensive overview of the experimental procedure. The process initiated with the preparation of loose and dry soil. The amount of cement to be added was based on the dry mass of the soil, utilizing percentages of 3%, and 6%, as presented in Table 3. The addition of GG, CLW or GY (as applicable) was also computed for the dry mass of the soil with 15% or 30% for CLW and 10% or 20% when GG and GY were added. The details of the mixed proportions compacted blends are explained in Table 3. Subsequently, the necessary quantity of water was added to attain the optimal moisture content of 18% in the mixture. Depending on the specific case, the moistened soil mixture was divided into three equal portions, each essential for compacting a single specimen and achieving the targeted dry unit weights (*γ*_d_) of 17 kN/m^3^, 17.6 kN/m^3^ and 18 kN/m^3^, as elucidated in Figure 4. The maximum dry unit weight from the Proctor standard was 17.6 kN/m^3^, and the optimum moisture content was 18%. The dry unit weight of molding was varied to study the effects of porosity on microstructural, compression and stiffness properties. The oven-drying method involved extracting a small sample from the separated mixture to ascertain whether it reached the desired moisture content.

The specimens underwent compaction in three layers, each possessing equal weight and density. Scarification was applied to the soil surface to ensure homogeneity between adjacent layers (Layers 1 and 2). The specimen was extracted from the mold, weighed and measured upon completion of compaction. All samples were compacted to a consistent volume of 196.35 cm^3^, utilizing a cylindrical mold with specific dimensions: 5 cm in diameter and 10 cm in height. Following compaction, the specimen was carefully wrapped in plastic, sealed and placed in a humid chamber for curing periods ranging from 7 to 27 days. Upon completion of the curing period, specimens were submerged in water to attempt saturation and minimize suction effects. Stringent quality acceptance criteria were implemented, stating that the maximum dry density should not deviate by more than 1% from the target dry unit weight. Similarly, the moisture content was required to exhibit no more than a 0.5% variance from the target moisture content. Curing occurred at a controlled environment of 23 °C with a relative humidity of 95% [35].

### 2.3. UCS and Stiffness (Non-Destructive) Program

Upon completion of the curing duration, the soil–cement–residue specimens were carefully removed from the humid chamber. A thorough re-weighing of the specimens followed the meticulous removal of the plastic wrapping. Subsequently, the specimens underwent immersion in distilled water, a deliberate step aimed at partially alleviating matric suction. This precautionary measure was taken to mitigate the potential impact of matric suction on the unconfined compressive strength, recognizing its significant influence on the mechanical properties of the specimens. The recommended and applied immersion duration was 24 h, as demonstrated in previous studies [28,36,37]. After immersion, the specimens were placed vertically within an automatic press for unconfined compression testing. The press had a sensitivity of 2.6 Newtons and operated at a speed of 1.15 mm per minute. The testing procedure followed the American standard ASTM D2166-03 [38]. Small pieces were taken from the tested specimens to determine the matric suction values of the samples. Several studies have related unconfined compressive strength (*q*_u_) to the porosity/cement index [39,40,41,42]. The porosity/cement index and *q*_u_ were adjusted through a power relation, as shown in Equation (1).

**Figure 5:**
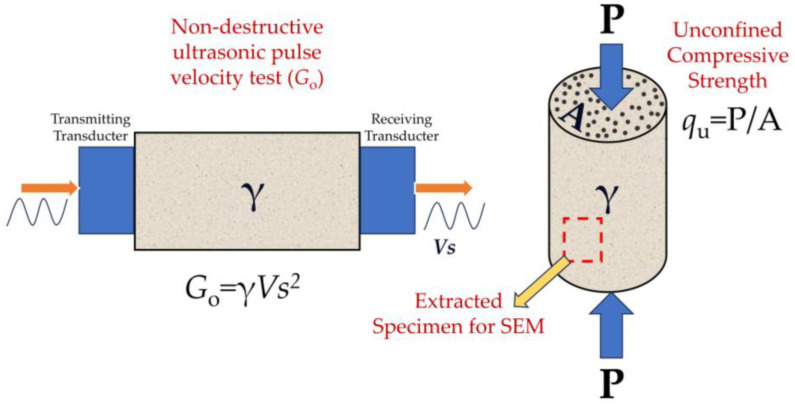
Test set-up of stiffness and unconfined compressive strength.

The stiffness or small-strain shear modulus (*G*_o_) was estimated using ultrasonic waves with Pundit Lab Plus equipment. Essentially, this equipment measures the propagation velocity of compression waves (P-waves) and shear waves (S-waves) in the material under testing. The first wave, a compression wave, was induced by the vibration of transducers at a frequency of 54 kHz, and the shear wave was obtained through the vibration of transducers at 250 kHz. The test set-up of stiffness and unconfined compressive strength is presented in Figure 5, where P is the rupture load during unconfined compressive tests and *A* is the cross-sectional area of the specimen. Notably, in non-dispersive media, the propagation time of the shear wave is independent of the employed frequency. The small-strain shear modulus of the material was obtained by multiplying the square of the shear wave velocity by the density of the material (Figure 5). Cylindrical specimens with a diameter of approximately 5 cm and an approximate height of 10 cm were used. This test was conducted in accordance with the ASTM D2845 standard [43].

### 2.4. Microstructural Analysis

SEM (scanning electron microscope) compositional analyses were meticulously carried out on carefully selected points within the sample specimens. Surface inspections of the compacted blends (Figure 5) were conducted at high magnifications of up to 2000 times, offering an incredibly detailed perspective of the material. Furthermore, for a more in-depth exploration of the distinctive features exhibited by the hydrated calcium crystals, we pushed the boundaries even further by employing magnifications of up to an astonishing 5000 times. These analytical investigations were expertly executed using the cutting-edge LIRA-3 instrument, Dual Beam SEM-FIB of TESCAN (Tescan Osay Holding, Brno-Kohoutovice, Czech Republic, made available to us by the esteemed Universidad de Los Andes in Colombia, ensuring the precision and reliability of our SEM tests. The SEM had an integrated microanalysis system using X-ray energy dispersion spectroscopy, EDX (Energy Dispersive X-ray Spectroscopy, Oxford XMAX 80, Aztec Software SP1 3.3 Nanoanalysis, Wiesbaden, Germany), which allowed obtaining a spectrum of the different chemical elements that the analyzed area may have contained and a relative percentage of each. Quantitative analyses had a precision of ±2% with detection limits of 100 ppm.

## 3. Results and Discussions

### 3.1. Effects of Porosity/Cement Index and Curing Periods on Strength for Soil–CLW–Cement Blends

**Figure 6:**
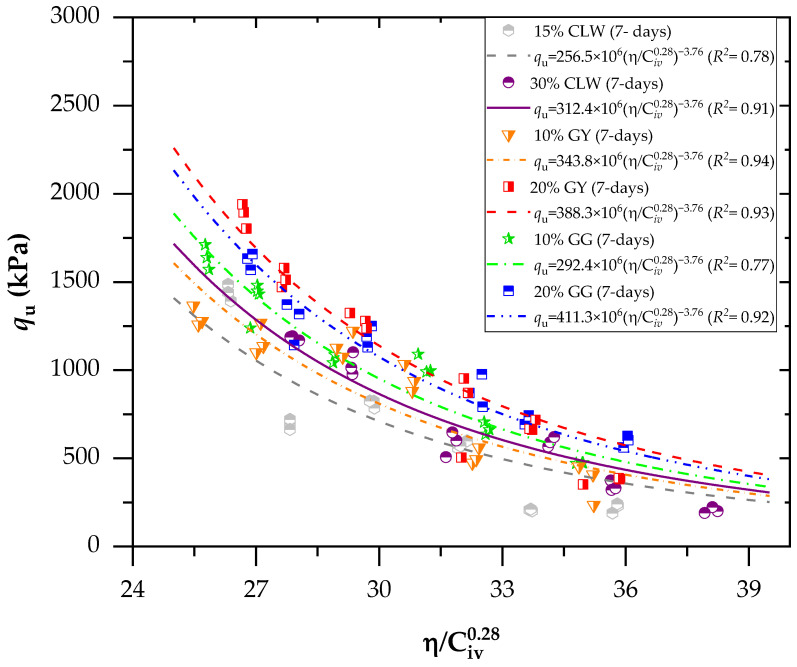
Effects of the porosity/cement index on the unconfined compressive of the new geomaterials cured after 7 days.

Figure 6 illustrates the relationship between the *η*/C*_iv_* index and the unconfined compressive strength of the soil mixture with CLW (crushed limestone waste), GY (gypsum) and GG (ground glass), considering a curing period of 7 days. To harmonize the unconfined compressive strength (*q*_u_) with the *η*/C*_iv_* factor, the volumetric index C*_iv_* had to be adjusted to an exponent of 0.28, calculated considering the data of the present study. Comparable to this, in some studies, the exact value has been calculated for various soil stabilizations [28,36,44]. The 0.28 exponent (less than 1) indicates that porosity exerted a more significant influence on the compressive strengths than the volumetric content of the binder [45].

In accordance with Figure 6 (considering a curing period of 7 days), for mixes stabilized with CLW, the increase in strength at 7 days of curing was 22% (comparing the addition of 15% to 30% CLW). Compared to a 10–20% addition, the corresponding increase for GY was 13%. Finally, by increasing the percentage of GG (ground glass) from 10 to 20%, there was a strength increase of 40%. Thus, the most significant increase in unconfined compressive strength (*q*_u_) occurred with recycled glass, the most reactive powdered material within the soil–cement mixture. However, adding 10% GY accelerated the reaction in the soil–cement mixture by more than 10% GG (ground glass). This was attributed to the fact that gypsum can act as a catalyst. The excellent suitability of the unconfined compressive strength (*q*_u_) concerning *η*/C*_iv_* can be discerned by observing the derived coefficient of determination (*R*^2^) of equations, which yielded values between 0.77 and 0.94. Unconfined compressive results were used to perform a statistical ANOVA, which included an analysis of five control factors: cement content; CLW, GG or GY content; dry unit weight; *η*/C*_iv_*; and curing period. The five factors significantly influenced the response variables (*p*-values below the 0.02 significance level) for *q*_u_, demonstrating the model’s suitability.

**Figure 7:**
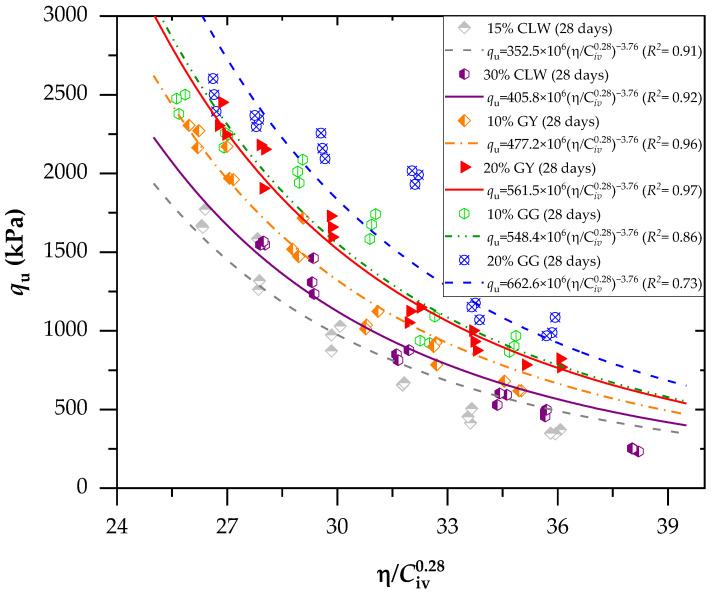
Effects of the porosity/cement index on the unconfined compressive strength of the new geomaterials cured after 28 days.

In accordance with Figure 7 (considering a curing period of 28 days), as observed, excellent dosage adjustments were generally obtained using the *η*/C*_iv_* method. For each adjustment, the equations used in predicting the mechanical behavior of mixtures of clay–cement with CLW, GG and GY residues were obtained. All equations reported in Figure 6 and Figure 7 follow the form of *q*_u_ = A(*η*/$C_{\mathrm{iv}}^{0.28})$^−B^. The A value increases when residues are increased in the mixture, and it increases when the curing time varies from 7 to 28 days. Recently, studies have simulated the relationship of Equation (1) with *η*/C*_iv_*. Baldovino et al. [45] used silt soil from the Guabirotuba formation in Brazil to stabilize with cement using 7, 28 and 90 days of curing and three levels of compaction efforts. The authors concluded that the *η*/C*_iv_* index is the main factor influencing the unconfined compressive strength of stabilized silt. Ekinci et al. [40] verified parameters controlling the unconfined compressive strength and loss of mass of reinforcement clay with fibers. The authors found that the porosity/cement index directly influences accumulated mass and strength loss.

### 3.2. Effects of Porosity/Cement Index and Curing Periods on Stiffness for Soil–CLW–Cement Blends

**Figure 8:**
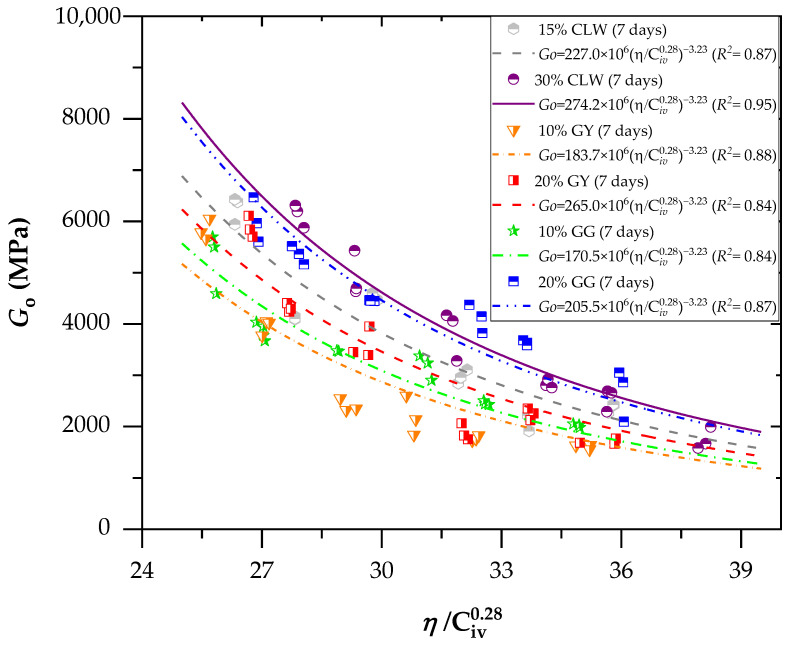
Effects of the porosity/cement index on the stiffness (*Go*) of the new geomaterials cured after 7 days.

**Figure 9:**
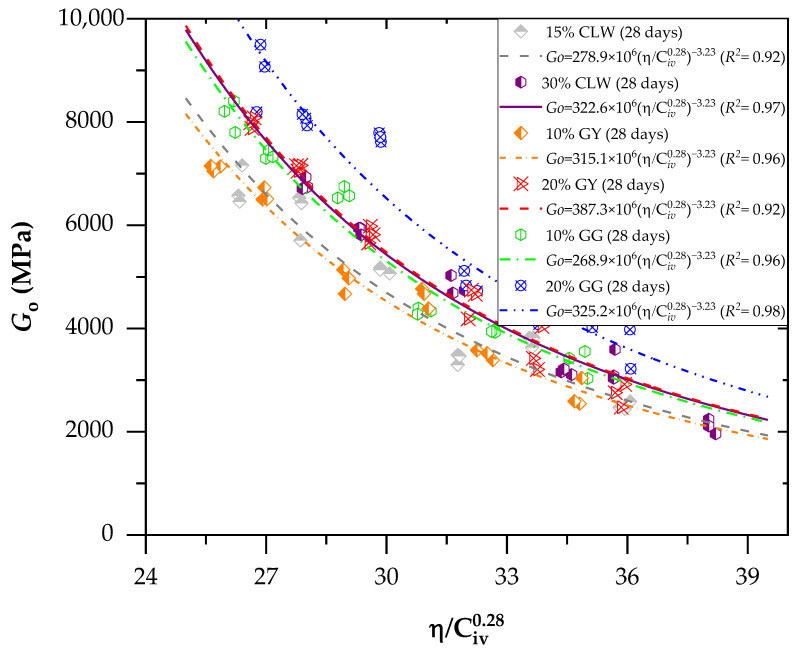
Effects of the porosity/cement index on the stiffness (*G*_o_) of the new geomaterials cured after 28 days.

Figure 8 and Figure 9 present the effects of the porosity/cement index on the stiffness (*G_o_*) of the new geomaterials cured after 7 and 28 days, respectively. For stiffness (*G_o_*), the porosity/cement ratio (adjusted to 0.28) varied from 25% to 38% for 7 and 28 days. Similarly, the stiffness increased with the curing time and reduced the sample porosity for unconfined compression. Through Equation (2), which relates the initial shear modulus (*G_o_*) to the parameter *η*/C*_iv_*, it is possible to observe an excellent fit of the method using the porosity/cement volumetric content index to predict the modulus *G_o_*. This result is demonstrated primarily by the coefficients of determination (*R*^2^) above 0.84 for the age of 7 days (Figure 8) and above 0.92 for the age of 28 days (Figure 9). The *G_o_* results were used to perform a statistical ANOVA. The five factors (as used in Section 3.1) significantly influenced the response variables (*p*-values below the 0.04 significance level) for *q*_u_, demonstrating the model’s suitability.

The equations in Figure 8 and Figure 9 represent the power curve that relates the modulus *G_o_* to the values of *η*/C*_iv_* for ages of 7 and 28 days, considering the additions of various residues: GG, GY and CLW.

(2) $$G_{o}=A{\left[\frac{\eta }{{\left(C_{iv}\right)}^{x}}\right]}^{-B}$$

Within each *η*/C*_iv_* value, minor differences in the average small strain stiffness were discernible across the tested dosages. These distinctions became more noticeable when the *η*/*C*_iv_ value reached 32%. However, despite these variations, a robust correlation between *G_o_* and the adjusted *η*/C*_iv_* index persisted, irrespective of the chosen mix design. Consequently, the contact area between particles maintained a comparable order of magnitude for the same adjusted *η*/C*_iv_* value. In simpler terms, in these specific scenarios, the increase in contact area due to a higher degree of cementation (in specimens with increased cement content) and *η* had an impact similar to that of the enlargement in contact area resulting from a greater degree of interlocking in less porous specimens with a small amount of cement. As a result, the initial shear modulus remained within a similar order of magnitude for each porosity/cement index value, regardless of the dosage employed. The cement improved various soil properties, increasing strength, bearing capacity, durability and stiffness; controlling expansion; reducing plasticity; and modifying the particle size distribution, as observed in various studies available in the literature [46]. Studies have emphasized that the initial stiffness may decrease when confinement stresses are high, leading to a transition from a dilatant and brittle behavior to a compressible and ductile behavior [47].

### 3.3. Normalization of Strength and Stiffness

According to Consoli et al. [48], it is possible to demonstrate the behavior of the curves of unconfined compressive strength and tensile strength versus the parameter *η*/$C_{\mathrm{iv}}^{0.28}$ in a single curve by normalizing these values. Normalization is performed by dividing the unconfined compressive strength (*q*_u_) or tensile strength (*q*_t_) by the strength value that the material would have for a fixed value of *η*/$C_{\mathrm{iv}}^{0.28}$, within the range obtained in the curves adjusted by the power function as defined by Equation (3). In the same way, Equation (4) shows the general equation for the normalization values of the stiffness *G_o_*.

(3) $$\frac{q_{u}}{q_{u-n}\left(\eta /C_{\mathrm{iv}}^{x}=\Delta \right)}=\frac{A{\left(\eta /C_{\mathrm{iv}}^{x}\right)}^{-B}}{A{\left(\Delta \right)}^{-B}}=\Delta^{B}{\left(\eta /C_{\mathrm{iv}}^{x}\right)}^{-B}$$

(4) $$\frac{Go}{Go-n\left(\eta /C_{\mathrm{iv}}^{x}=\Delta \right)}=\frac{A{\left(\eta /C_{\mathrm{iv}}^{x}\right)}^{-B}}{A{\left(\Delta \right)}^{-B}}=\Delta^{B}{\left(\eta /C_{\mathrm{iv}}^{x}\right)}^{-B}$$

**Figure 10:**
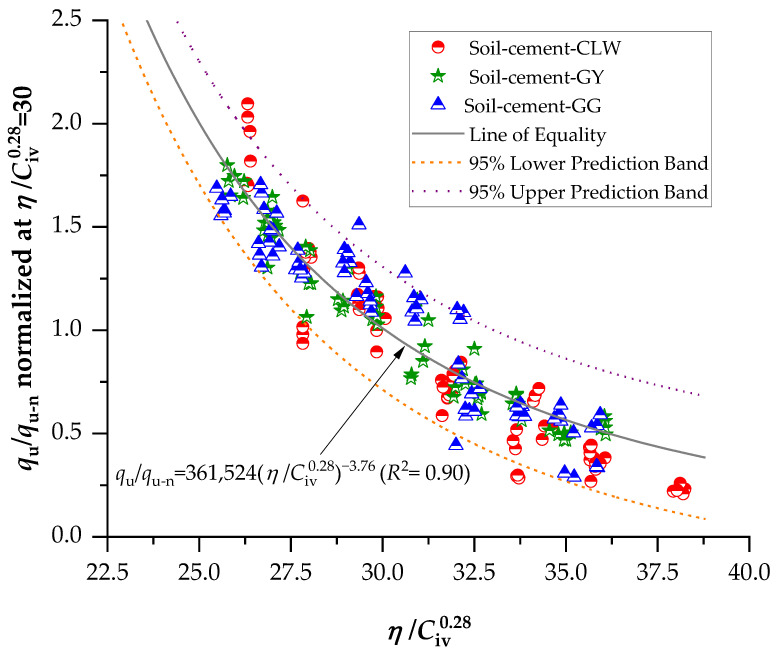
Normalization of unconfined compressive strength using the porosity/cement index.

To normalize the curves in the present study and apply Equations (3) and (4) for *q*_u_ and *G*_o_, respectively, the value of *η*/$C_{\mathrm{iv}}^{0.28}$ = 30 was chosen; this value was adopted arbitrarily, but it is emphasized that this value is within the range of *η*/$C_{\mathrm{iv}}^{0.28}$ values obtained during molding. Figure 10 shows the curve of normalized values of *q*_u_ for the chosen value. It can be observed that the normalization model proposed by Consoli et al. [48] is suitable for the tests conducted on the soil–cement stabilized with different residues (GG, GY and CLW), representing *q*_u_ in Equation (5) presented below, through a power function that yields an *R*^2^ of 0.90.

(5) $$\frac{q_{u}}{q_{u-n}\left(\eta /C_{\mathrm{iv}}^{0.28}=30\right)}=361,524{\left(\eta /C_{\mathrm{iv}}^{0.28}\right)}^{-3.76}\;(R^{2}=0.90)$$

**Figure 11:**
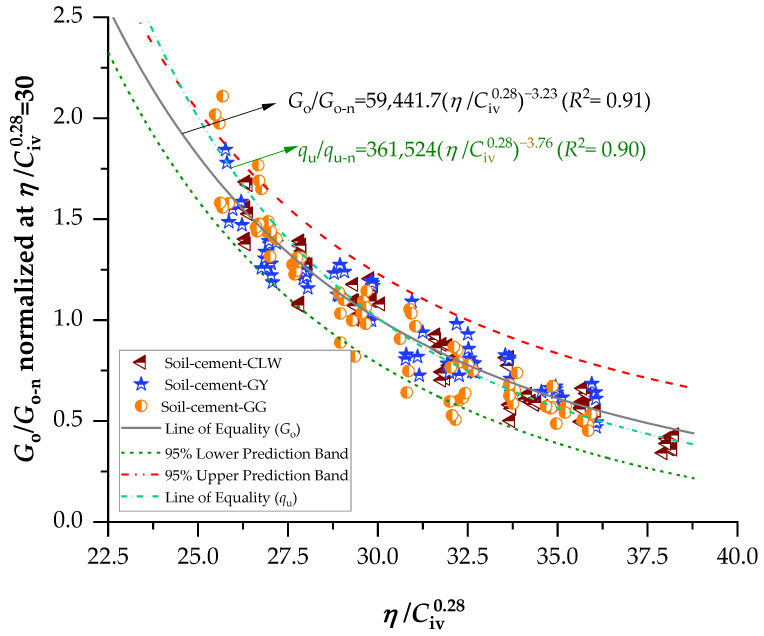
Normalization of stiffness data using the porosity/cement index.

The same data treatment was applied to the initial shear modulus *G_o_*, normalizing its values by dividing them by a determined *G_o_* value for the same index *η*/$C_{\mathrm{iv}}^{0.28}$ = 30. Figure 11 shows the graph plotted from the results, fitted by the power equation presented below (Equation (6)), with a coefficient of determination (*R*^2^) equal to 0.91. It is noted that, as it involves adjusting the same index as that used to normalize *q*_u_ and *G_o_*, the resulting formulations were the same, indicating that the previous normalization is also valid for the initial shear modulus.

(6) $$\frac{G_{o}}{G_{o-n}\left(\eta /C_{\mathrm{iv}}^{0.28}=30\right)}=59,441.7{\left(\eta /C_{\mathrm{iv}}^{0.28}\right)}^{-3.23}\;(R^{2}=0.91)$$

**Figure 12:**
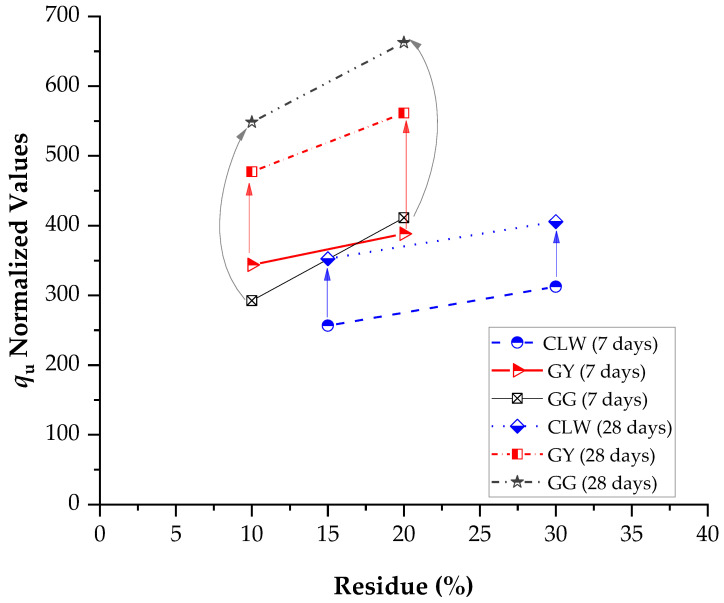
Main effects of CLW, GY, GG and curing times on unconfined compressive compacted blends.

**Figure 13:**
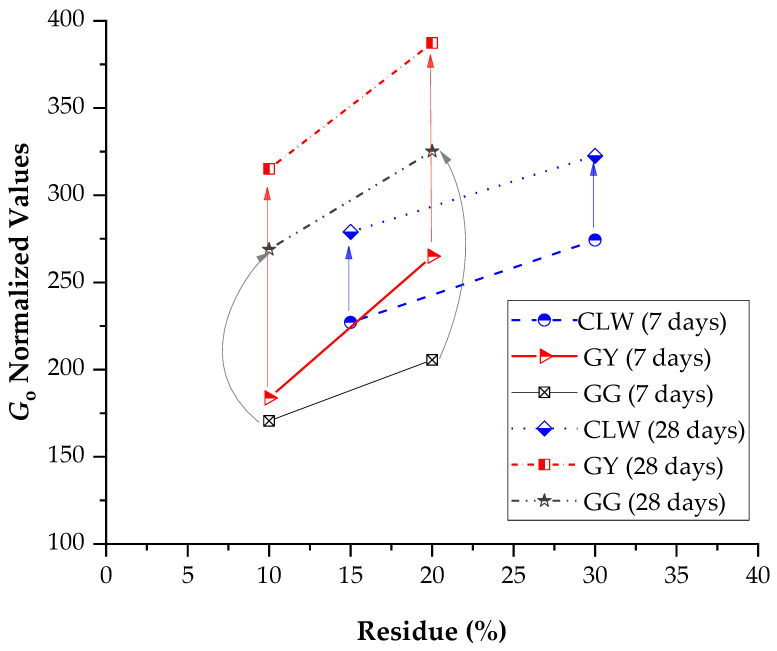
Main effects of CLW, GY, GG and curing times on stiffness of compacted blends.

Figure 12 presents the normalized results in terms of the constant A (Equation (1)), used to calculate the influence of the percentages of residues on the unconfined compressive strength. For 7 days of curing, the highest strength was developed by stabilized soil–gypsum mixes, followed by soil–GG mixes and last by soil–CLW mixes. Previous studies have shown that gypsum accelerates the strength of soil–cement development in a short curing time [49,50,51]. For 28 days of curing, the soil–glass samples achieved the highest strengths, followed by soil–gypsum, and the samples that developed the most minor strength were those of soil–CLW. Figure 13 presents the main effects of CLW, GY and GG on stiffness considering 7 and 28 days of curing. Similar to strength, stiffness increases with the percentage of residues. This includes the modulus of elasticity, dry unit weight, shear modulus and thermal properties. These are just some properties that can influence wave propagation speed in a specific material. The exact relationship may vary depending on the type of wave (e.g., longitudinal or shear waves) and the type of material.

**Figure 14:**
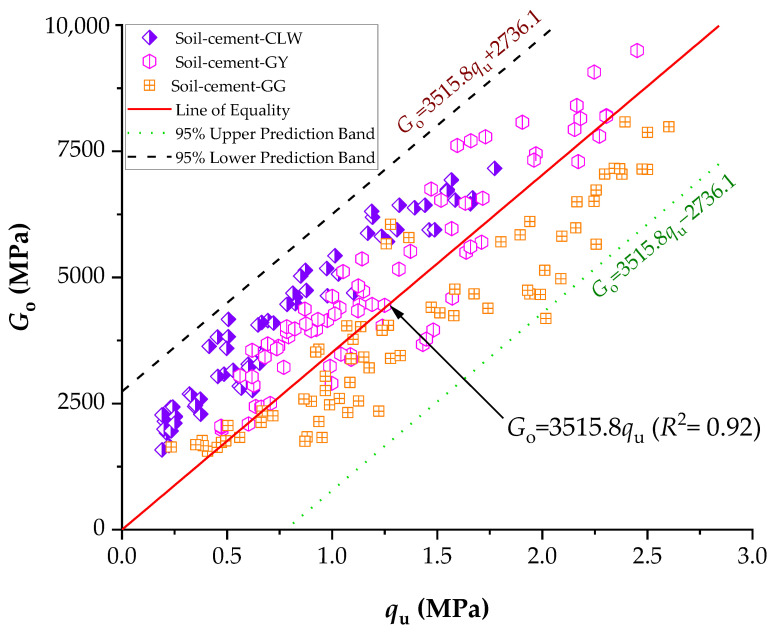
Direct relationship between the UCS and stiffness.

The stiffness data have a consistent pattern similar to the results obtained from the unconfined compression tests (Figure 14). Specifically, there is an inverse correlation when linked to the *η*/$C_{\mathrm{iv}}^{0.28}$ index, and likewise, there are slight variations among distinct dosages with the same adjusted porosity/cement value. Consequently, there is a direct relationship between the strength and stiffness of the specimens under study, as illustrated in Figure 14. In summary, samples with greater stiffness also tend to have higher strength values. One linear relationship emerged when correlating the initial shear modulus data with the outcomes of unconfined compressive strength (i.e., *G_o_* = 3515.8*q*_u_). For two zones, each corresponding to a specific *η*/$C_{\mathrm{iv}}^{0.28}$, the value was identifiable (+2736.1 or −2736.1). The distinction between the two linear trends is crucial for understanding the mechanical behavior of soil–cement specimens. The first trend, characterized by passing through the origin, implies a direct correlation between the compacting process and the absence of unconfined compressive strength. In practical terms, this means that a soil–cement mixture compacted under these conditions would not only lack unconfined compressive strength but also exhibit a notable absence of rigidity at small strain values. It can be observed in Figure 13 and Figure 14 that the material addition (CLW, GY and GG) changed the small strain stiffness of the clay soil measured, again confirming that cement and raw material additions affect the behavior at small strains. Also, it can be noticed that, at very small strains, the three types of compacted blends were coincident, as shown in Figure 14, confirming the ultrasonic test data of observations from the curves presented in Figure 9.

On the other hand, the second linear trend, not originating from the origin, introduces a different dynamic. In this interpretation, the sample displays stiffness even when unconfined compressive strength is absent. This suggests a more nuanced relationship between the compacting process and the development of rigidity at small strain values. The departure from the origin implies that other factors or mechanisms might contribute to the stiffness observed, even when traditional strength measures are absent. In summary, these distinct interpretations highlight the complexity of the relationship between compacting conditions, unconfined compressive strength and the rigidity of soil–cement specimens. The first interpretation underscores a direct connection between compaction and the absence of strength, and the second allows for stiffness even in the absence of traditional strength measures, indicating the presence of additional contributing factors.

### 3.4. Microstructure and Microanalysis of Compacted Blends

**Figure 15:**
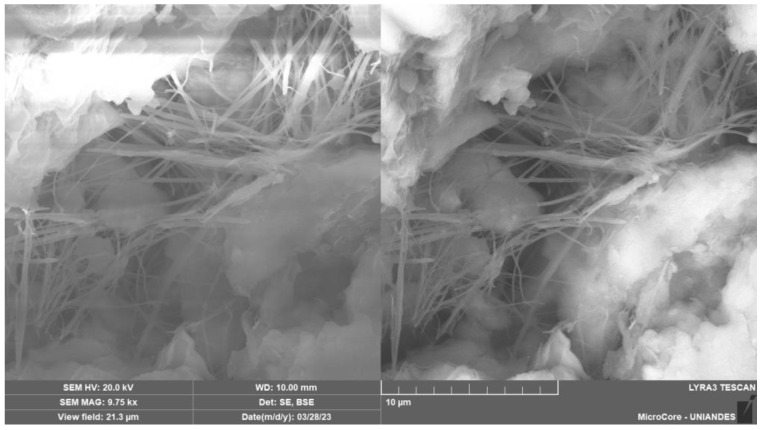
SEM micrographs of the CLW–soil–cement compacted blend.

**Figure 16:**
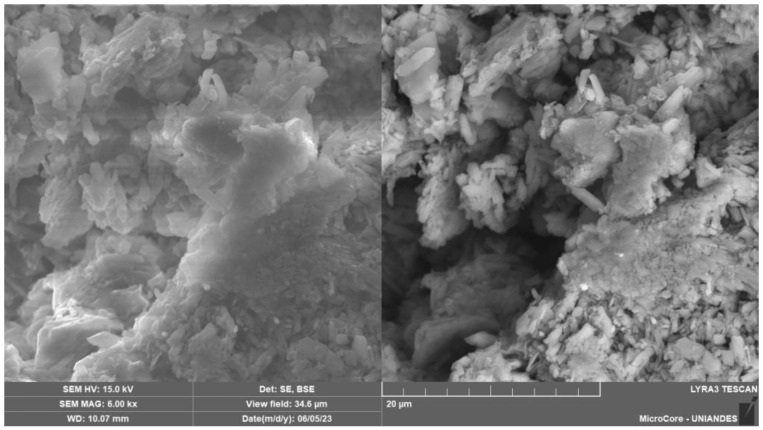
SEM micrographs of the recycled gypsum plaster–soil–cement compacted blend.

**Figure 17:**
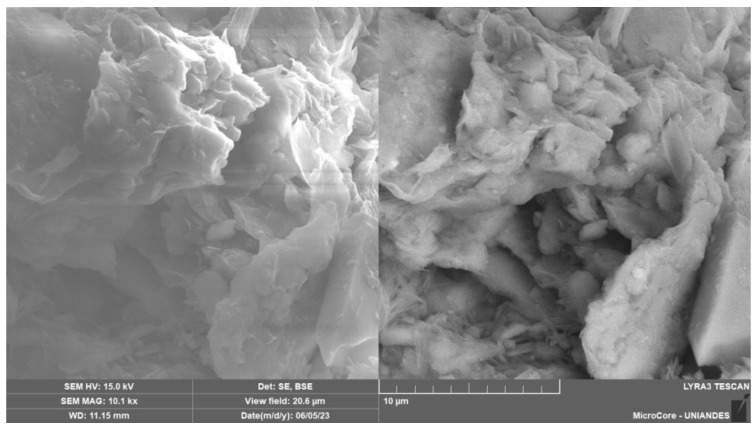
SEM micrographs of the ground glass–soil–cement compacted blend.

Figure 15, Figure 16 and Figure 17 provide the SEM micrographs of the soil–cement compacted blends with CLW, GY and GG, respectively. High silica, oxygen and aluminum levels were found in the natural soil components (Figure 3a and Table 2). The same components were present in the fine fraction, and there was more calcium due to the clay minerals contributing more calcium (Figure 15, Figure 16 and Figure 17). In the cement, high contents of silica, calcium, oxygen, carbon and iron were found (Table 2). Mineralogical changes in the curing times of the soil–cement mixtures (with CLW, GY or GG) were found, as shown in Figure 15, Figure 16 and Figure 17. The cement particles bound to those of the soil. The inter-aggregate pores were reduced. In SEM images, gelifications presented as bridges due to the action of the cement.

Figure 15 shows the appearance of elongated tubes similar to ettringite formed in the soil–cement mixture. Ettringite [Ca_6_Al_2_(SO_4_)_3_(OH)_12_·26H_2_O] is a hydration of the C_3_A product in cement due to the natural process of combining cement, water and calcium sulfate. The growth of these cement reaction products made the soil structures denser, increasing strength as represented by the decrease in the *η*/C*_iv_* function (Figure 6 and Figure 7).

Several distinct features were observed in the SEM micrographs of the recycled gypsum compacted with the soil–cement blend, as shown in Figure 16. First, there was a noticeable agglutination of crystals, indicating a tendency for these particles to cluster together. Additionally, the formation of large nuclei was evident, a sign of the coalescence of smaller particles into larger, more prominent groupings. Moreover, the micrographs revealed an intricate entanglement of the CaSO_4_ crystals, showcasing a complex interweaving pattern typical of this material’s microstructural behavior. From the SEM images of the soil–cement–GY compacted blends, one can observe the formation of agglomerates consisting of elongated microcrystals. These microcrystals exhibited varying dimensions and were notably dispersed throughout the mixture of soil and cement. This observation suggests an interaction between the gypsum microcrystals and the other components in the mix, forming these unique agglomerates.

The manifestation of densified C-S-H (calcium-silicate-hydrate) structures is depicted in Figure 17. This phenomenon is particularly notable when the mix is augmented with increased amorphous silicon. The presence of this additional silicon markedly enhances its interaction with the hydration products of the blend. This interaction catalyzes the formation of more robust C-S-H structures [51]. As a result, these structures contribute to a more substantial confinement within the pore matrix of the material. Similar results were obtained by Baldovino et al. [52] when analyzing silty soil with lime and glass powder, finding C-S-H and ettringite factories hardened on the surface of the stabilized soil in the early stages of curing. Consequently, there was a noticeable reduction in the size and number of capillary channels within the composite. This decrease in capillary porosity implies a denser material formation and suggests an improvement in the structural integrity and, potentially, the composite’s durability. Such changes are vital to understanding the mechanical and permeability characteristics of the developed material.

Considering the size of GG used in the present study (smaller than 0.12 mm), Jani and Hogland [53] highlighted that the pozzolanic characteristics of finely ground glass are significantly amplified when the particles are smaller than 100 μm, comparable to the dimensions employed in this investigation. This becomes particularly evident when this material partially substitutes Portland cement in concrete mixtures. Complementing this perspective, Omran et al. [18] analyzed the efficacy of concrete mixed with glass powder. They proposed that the principal advantages of integrating crushed glass into concrete manifest in the improved microstructural composition, the sustained reduction in pore size within the concrete over time and the overall enhancement of its durability characteristics.

## 4. Conclusions

- The 7-day curing period results indicate significant strength improvements in CLW-stabilized mixes (22% increase from 15% to 30% CLW), GY-stabilized mixes (13% increase from 10% to 20% GY) and GG-stabilized mixes (40% increase from 10% to 20% GG). GG showed the most substantial enhancement in unconfined compressive strength, highlighting its reactivity in the soil–cement mixture. Moreover, adding 10% GY accelerated the reaction more than 10% GG, underscoring gypsum’s catalytic role. The strong correlation between unconfined compressive strength and *η*/$C_{\mathrm{iv}}^{0.28}$ is evident, with *R*^2^ values ranging from 0.77 to 0.94, emphasizing the reliability of *q*_u_ concerning void content relative to binder quantity.
- This research introduces promising materials for stabilizing clayey soils, particularly from northern Colombia. The research introduces alternatives: crushed ground glass (GG), recycled gypsum (GY) and waste from limestone quarry crushing (CLW), which are construction byproducts. Testing revealed a direct correlation between compressive strength (*q*_u_), initial stiffness (*G_o_*) and porosity/cement index *η*/$C_{\mathrm{iv}}^{0.28}$, providing valuable insights into the performance of these novel geomaterials.
- The SEM micrographs in Figure 15, Figure 16 and Figure 17 reveal critical structural changes in soil–cement blends with CLW, GY and GG. Figure 15 shows hydration product formation, densifying soil for increased strength. Figure 16 highlights crystal agglutination, nuclei formation and entanglement of CaSO_4_ crystals in recycled gypsum, suggesting unique agglomerates. Figure 17 depicts densified calcium-silicate-hydrate structures, implying improved structural integrity and reduced porosity. These findings offer valuable insights into the mechanical and permeability characteristics of the developed materials.

## Figures and Tables

**Table 1:** Characteristics and properties of the soil sample, CLW, GG and GY. NP, non-plastic. ML, inorganic silt. SW, well-graded sand. CL, inorganic clay.

Property	Standard/Reference	Unit	Value			
			Soil	CLW	GG	GY
Limit Liquid, L.L.	[31]	%	42.0	-	-	-
Plasticity Index, P.I.	[31]	%	15.9	NP	NP	NP
Specific Gravity, Gs	[32]	-	2.80	2.52	2.40	2.33
Gravel (4.75–76.2 mm)	[33,34]	%	0	10	0	0
Coarse Sand (2.00–4.75 mm)	[33,34]	%	0	30	0	0
Medium Sand (0.425–2.0 mm)	[33,34]	%	0	38	0	6
Fine Sand (0.075–0.425 mm)	[33,34]	%	8	17	13	59
Silt (0.002–0.075 mm)	[33,34]	%	82	15	83	32
Clay (<0.002 mm)	[33,34]	%	10	0	4	3
Mean Diameter (*d*_50_)	[33,34]	mm	0.011	1.6	0.016	0.98
Effective Diameter (*d*_10_)	[33,34]	mm	0.0021	0.15	0.0035	0.007
Uniformity Coefficient *C*_u_	[33,34]	-	7.14	13.67	5.71	18.50
Coefficient of Cuvature *C*_c_	[33,34]	-	0.96	1.59	1.03	1.76
Activity of Clay	[30]	-	1.60	-	-	-
USCS Classification	[33]	-	CL	SW	ML	SW
Color	Munsell Chart	-	Black	Gray	White	White

**Table 2:** Chemical composition of the soil sample, cement, CLW, GG and GY.

Compound	Chemical Contents by Weight (%)				
	Soil	Cement	CLW	GG	GY
CaO	3.0	62.7	72.4	7.1	50.5
MgO	-	3.8	2.1	2.2	-
SiO_2_	66.0	21.1	9.0	78.2	0.4
Al_2_O_3_	21.1	5.2	1.3	2.2	-
Fe_2_O_3_	0.9	2.6	0.9	0.19	0.3
TiO_2_	0.3	-	-	-	-
K_2_O	3.1	-	-	-	-
SO_3_	4.0	3.5	-	-	37.6
Na_2_O	-	0.1	-	9.3	1.3
MnO	-	0.2	14.3	-	-
P_2_O_5_	-	-	72.4	-	1.9
LOI	1.6	0.8	2.1	0.7	8.0

**Table 3:** Mixed proportion design for compacted blends of soil, cement, CLW, GG and GY.

Mix	Weight (%)					Curing Times (d)	Molding *γ*_d_ (kN/m^3^)	Number of Specimens
	Soil	Cement	CLW	GG	GY			
Soil–cement–CLW	100	3	15	-	-	7, 28	17, 17.6, 18	18
	100	3	30	-	-	7, 28	17, 17.6, 18	18
	100	6	15	-	-	7, 28	17, 17.6, 18	18
	100	6	30	-	-	7, 28	17, 17.6, 18	18
Soil–cement–GG	100	3	-	10	-	7, 28	17, 17.6, 18	18
	100	3	-	20	-	7, 28	17, 17.6, 18	18
	100	6	-	10	-	7, 28	17, 17.6, 18	18
	100	6	-	20	-	7, 28	17, 17.6, 18	18
Soil–cement–GY	100	3	-	-	10	7, 28	17, 17.6, 18	18
	100	3	-	-	20	7, 28	17, 17.6, 18	18
	100	6	-	-	10	7, 28	17, 17.6, 18	18
	100	6	-	-	20	7, 28	17, 17.6, 18	18

## Data Availability

Data are contained within the article.
